# Effect of Aerobic Exercise and a Diet Supplementation with Linoleic Acid on Metabolic Parameters in *Drosophila melanogaster*

**DOI:** 10.3390/biology15080607

**Published:** 2026-04-12

**Authors:** Thiago Henrique Oliveira Alves, Jadyellen Rondon Silva, Ingrid Mendes Limeira, Samantha Rhein, Anderson Oliveira Souza

**Affiliations:** 1 Mitochondrial Metabolism and Neurotoxicology Laboratory, Department of Chemistry, Institute of Chemistry, Federal University of Mato Grosso, Cuiabá 78060-900, Brazil; thiagohenrrique.henrrique@gmail.com (T.H.O.A.); jrondon586@gmail.com (J.R.S.); limeira0mendes@gmail.com (I.M.L.); 2 Institute of Health Sciences, O’Higgins University, Rancagua 28200-000, Chile; samantha.rhein@uoh.cl

**Keywords:** *Drosophila melanogaster*, biochemical and behavioral parameters, linoleic acid, muscle metabolism, physical exercise

## Abstract

Modern lifestyles characterized by reduced physical activity and changing eating habits have contributed to a global rise in obesity. This research examined the effects of a diet rich in linoleic acid combined with physical exercise using a TreadWheel system in *Drosophila melanogaster*. The flies were fed diets with varying linoleic acid concentrations from the larval stage through to day 15 of adulthood. A diet containing 45.9 mg/mL of linoleic acid improved eclosion rates, body weight, and biochemical markers such as glycogen, cholesterol, and hydrogen peroxide levels, as well as citrate synthase and acetylcholinesterase activities in sedentary flies. Conversely, flies that consumed linoleic acid and underwent 15 days of exercise on the TreadWheel showed increased weight, lactate, glycogen, cholesterol, nitric oxide levels, and acetylcholinesterase activity. These results suggest that a 15-day regimen of linoleic acid intake combined with physical exercise on the TreadWheel enhances muscle parameters in *D. melanogaster*, serving as an alternative animal model for nutrition and exercise research.

## Simple Summary

Modern lifestyles, with lower physical activity and changing eating habits, have caused a worldwide increase in chronic diseases linked to metabolic health. A diet rich in polyunsaturated fatty acids, especially linoleic acid, which is essential for brain development, and regular exercise provide numerous health advantages. However, consuming too much linoleic acid can generate more inflammatory compounds, increasing the risk of serious heart problems. To explore how a diet high in linoleic acid and/or physical activity (TreadWheel) affects thoracic metabolism, we used *Drosophila melanogaster* as our animal model, a widely recognized species for nutrition and neuroscience studies.

## 1. Introduction

Senescence within the population has been rising over the past ten years, raising concerns about enhancing their quality of life due to the link between aging and functional decline, such as sarcopenia. Sarcopenia is a disease associated with low-grade inflammation, characterized by reduced muscle mass and quality, resulting in decreased strength, respiratory capacity, basal metabolic rate, muscle fat oxidation, peripheral insulin sensitivity, and other health issues that increase the risk of chronic non-communicable diseases (NCDs) [1,2]. To mitigate the risk of NCDs, physical exercise (PE) is recommended because it helps counteract these negative effects.

Regular PE induces structural and functional adaptations that confer health benefits, including enhanced muscle growth, increased bone density, reduced peripheral vascular resistance, and improved peripheral insulin sensitivity. It serves as a non-drug method for managing NCDs like hypertension, obesity, and type 2 *Diabetes mellitus*, conditions that can elevate the risk of neurodegenerative diseases (ND) [2,3,4,5].

Other risk factors include a pro-inflammatory profile associated with a sedentary lifestyle and central obesity. White adipose tissue, which contributes nearly one-third of IL-6 (Interleukin-6) production, is linked to faster muscle mass loss, a common problem in cancer patients. Besides the previously noted morphofunctional changes, PE may have anti-inflammatory effects by increasing IL-6 receptor expression, reducing circulating IL-6, and decreasing Toll-like receptor 4 (TLR-4) levels. TLR-4 plays a role in acute inflammation pathways triggered by extracellular pathogens like bacteria and fungi. However, overexpression of TLR-4 is associated with obesity and insulin resistance. Additionally, muscle releases anti-inflammatory interleukins such as IL-1, IL-10, and IL-5 during PE [6,7,8].

Maintaining a balanced diet and engaging in regular exercise provide numerous health benefits, including adequate intake of linoleic acid (LA), an essential polyunsaturated fatty acid (PUFA) found in plant sources such as corn. LA is crucial for central nervous system development, along with alpha-linolenic acid (ALA). Consuming foods rich in LA, such as corn oil, is part of a healthy diet. Additionally, the recent increase in processed food consumption likely raised the LA to ALA ratio, contributing to a pro-inflammatory state [9,10,11,12,13]. To explore how a diet high in LA and/or exercise affects metabolism, we used *Drosophila melanogaster*, a widely recognized model for studying neurodegenerative diseases [14,15,16,17,18], toxicology [19,20,21], and nutrition [22,23].

Studies involving PE in *Drosophila melanogaster* over three weeks showed increased mitochondrial activity [24]. Additionally, PE can elevate octopamine levels, a neurotransmitter in invertebrates linked to performance and exercise-related adaptations in *D. melanogaster*’s cardiac and muscular systems [25,26,27]. Moreover, assays with LA supplementation revealed a slowdown in disease progression in Alzheimer’s models and improved adaptive immune responses in *D. melanogaster* [28]. This research may inform recommendations for combining LA supplementation with physical activity, with an emphasis on behavioral, physiological, and biochemical effects in *D. melanogaster*.

## 2. Materials and Methods

### 2.1. Fly Strain and Rearing

All experiments in this study were performed using w^1118^ (wild-type) flies. The flies were cultured at 25 ± 1 °C on a standard diet containing cornmeal (6.5% *w*/*v*), agar (CAS n. 9002-18-00) (1.0% *w*/*v*), yeast (6.5%), and nipagin (CAS n. 99-76-3) (3.0% *v*/*v*) [29,30].

### 2.2. Exposure to a Diet Supplemented with Linoleic Acid

Both male and female flies were kept under four experimental conditions for 3 h. During this period, the adult’s flies were allowed to lay eggs for 3 h, and the larvae eclosed were fed: (1) a standard diet (SD) containing cornmeal, agar, yeast, and nipagin; (2) a SD supplemented with 45.9 mg/mL of linoleic acid (LA) by corn oil; (3) a SD supplemented with 37.9 mg/mL of LA; and (4) a SD supplemented with 18.9 mg/mL of LA (final concentration).

### 2.3. Determination of the Pupal Volume of Drosophila melanogaster

After five days, larvae fed different diets begin to metamorphose into adults. To evaluate the effect of the supplemented diet on the volume of *D. melanogaster* pupae, we measured the volume or axial ratio (length/width) of 40 pupae [31].

### 2.4. Exercise Protocol

To exercise the flies, we used a system called TreadWheel, which systematically inverts the fly vials (13 × 2.5 cm length × width) to stimulate negative geotaxis without lifting and dropping them. Exercise vials allowed the flies to move six centimeters, while the controls (sedentary flies) were placed in vials on the TreadWheel, limiting them to one centimeter of space during the exercise session. As a result, they experienced rotation with restricted mobility. All experiments were conducted for 120 min at 4 rpm [24,27] over a period of 15 days.

The flies (males and females) were divided into eight groups: (1) standard diet (SD) and TreadWheel exercise; (2) SD supplemented with 45.9 mg/mL of LA and TreadWheel exercise; (3) SD supplemented with 37.9 mg/mL of LA and TreadWheel exercise; (4) SD supplemented with 18.9 mg/mL of LA and TreadWheel exercise; (5) SD and sedentary TreadWheel; (6) SD supplemented with 45.9 mg/mL of LA and sedentary TreadWheel; (7) SD supplemented with 37.9 mg/mL of LA and sedentary TreadWheel; (8) SD supplemented with 18.9 mg/mL of LA and sedentary TreadWheel. The experiments were conducted in quintuplicate (80 flies per vial).

After 15 days on the TreadWheel, all females (50 thoraces/microtubes) were dissected and homogenized with tungsten carbide spheres (cat. n. 69997, Qiagen, Venlo, The Netherlands) in a solution (0.9% NaCl, pH 7.0) (CAS n. 7647-14-5) containing a protease inhibitor diluted 1:200 (CAS n. 66701-25-5). The homogenates were centrifuged at 10,000× *g* for 10 min at 4 °C. The supernatant was collected and stored at −20 °C until use.

### 2.5. Fly Weight

To analyze the weight gain of animals subjected to physical exercise for 15 days [24], FlyNap was used as an anesthetic. The results were calculated using the equation (final weight − initial weight) × number of flies per group.

### 2.6. Survival Assay

The effect of dietary supplementation and the TreadWheel apparatus on the lifespan of adult flies was evaluated using newly emerged flies. On the first day after eclosion, the flies were transferred to either a standard diet (control) or a diet supplemented with corn oil (120 flies per vial and 5 vials per group). The vials were changed every two days to provide fresh food, ensure optimal physiological conditions, and prevent mortality from causes like sticking to moist food, mold, or bacterial growth [32]. Mortality was recorded in each vial over a period of 15 days.

### 2.7. Lactate Production

Fifty thoraces (dissected and stored at −20 °C in the presence of a protease inhibitor) were macerated and homogenized in 0.1 M sodium phosphate buffer (CAS n. 7601-54-9), pH 7.4. The homogenate was centrifuged at 10000× *g* for 10 min at 4 °C, and the supernatant was collected [22]. Lactate content was measured using the lactate oxidase method, following the manufacturer’s instructions (Labtest, Lagoa Santa, Brazil, cat. no. #138-1/50). Absorbance was measured spectrophotometrically at 550 nm using a Model Varian Cary 50 MPR spectrophotometer (Varian Ltd., Melbourne, Australia). Lactate content was expressed as mg/dL of lactate per total protein content in the sample.

### 2.8. Glycogen Detection

*D. melanogaster* thoraces homogenates were boiled with 30% KOH (CAS n. 105033) for 20 min at 100 °C; then ethanol was added, and the mixture was centrifuged at 5000× *g* for 15 min at 4 °C. The supernatant was discarded, and a solution containing 0.2% antrone (CAS n. 102694653) was added to the pellet [33]. The samples were read at 612 nm using a Model Varian Cary 50 MPR spectrophotometer (Varian Ltd., Melbourne, Australia).

### 2.9. Total Cholesterol

The cholesterol level in the sample was measured using a kit (Bioclin, Belo Horizonte, Brazil, catalog number #K083).

### 2.10. Reduced Glutathione (GSH)

Thoraces of *D. melanogaster* were homogenized in 100 mM sodium phosphate buffer (pH 7.4) containing 6 mM EDTA and 1 mg/mL OPT for 15 min [34]. Glutathione levels were evaluated spectrophotometrically at 420 nm using a Model Varian Cary 50 MPR spectrophotometer (Varian Ltd., Melbourne, Australia).

### 2.11. Hydrogen Peroxide (H_2_O_2_) Production

Ferrous oxidation with xylenol orange (FOX) includes 0.1 mM of xylenol orange, 0.25 mM of ferrous ammonium sulfate, and 100 mM of sorbitol. A 10 μL sample is added to 190 μL of FOX reagent, mixed, and incubated at room temperature for at least 30 min [35]. The optical density is measured spectrophotometrically at 560 nm using a Model Cary 50 MPR spectrophotometer (Varian Ltd., Melbourne, Australia).

### 2.12. Nitric Oxide Production

The Griess A solution was adapted from Vargas-Maya et al. (2021) [36], in which 0.1% N-(1-naphthyl) dihydrochloride ethylenediamine (CAS n. 1465-25-4) prepared in Milli-Q water was used. Solution B: 1% sulfanilamide (CAS n. 63-74-1) prepared in 5% phosphoric acid (CAS n. 7664-38-2). For nitrite (CAS n. 7632-00-0) concentrations in the standard nitrite curve ranging from 0.5 to 20 μM, the final concentration was prepared in each experiment by measuring biological samples. The standard curve and analysis of the biological sample (fifty thoraces) were prepared by adding 50 μL of Solution A and 50 μL of Solution B and then added to a 96-well plate. Reactions were incubated at room temperature for 15 min, protected from light. Optical density was measured spectrophotometrically at 540 nm using a spectrophotometer (Model Cary 50 MPR Varian Ltd., Melbourne, Australia).

### 2.13. Citrate Synthase Activity

Fifty thoraces (dissected and stored at −20 °C in protease inhibitor) were subjected to homogenization in Tris (200 mM, pH 8.0) with Triton X-100 (CAS n. 9036-19-5) (0.2% *v*/*v*) [37]. Then, the homogenates were centrifuged at 9000× *g* for 30 min at 4 °C, and the supernatant was collected. The protein concentration was then determined. CS activity was initiated by the addition of 0.01 mg of protein to ~170 μL of Tris buffer containing 10 mM of acetyl-CoA (CAS n. 32140-51-5), 1 mM of 5′5′-Dithiobis-2-nitrobenzoic acid (DTNB) (CAS n. 69-78-3) and 10 mM of oxaloacetate (CAS n. 328-42-7). The reduced CoA (CoA-SH) formed by CS activity converts DTNB to 2-nitro-5-benzoic acid (TNB). CS activities were evaluated by the TNB formation rate, measured spectrophotometrically at 412 nm according to Srere (1969) [38] using a Varian Model Cary 50 MPR spectrophotometer (Varian Ltd., Melbourne, Australia).

### 2.14. Acetylcholinesterase (AChE) Activity

Fifty thoraces of *D. melanogaster* were homogenized in 100 mM sodium phosphate buffer (containing inhibitory protease), pH 7.4, to disrupt the cells. The homogenates were centrifuged at 9000× *g* for 30 min at 4 °C. The supernatant (0.01 mg protein) was incubated with 100 mM sodium phosphate buffer, pH 7.4, containing 150 mM acetylthiocholine (CAS n. 1866-15-5) and 1 mM DTNB. The AChE activity was determined spectrophotometrically using a Varian Model Cary 50 MPR spectrophotometer (Varian Ltd., Melbourne, Australia). The results were expressed as conjugate nmol formed/min/mg of protein [39].

### 2.15. Protein Quantification

Protein concentration was determined by the Bradford assay using BSA (CAS n. 9048-46-8) as a standard. The assay involves the interaction of the protein with the Coomassie Blue reagent (CAS n. 6104-59-2), with readings performed at 596 nm [40].

### 2.16. Statistical Analyses

The data are presented as an average ± SEM. N represents the number of female flies per group used in each experiment. Statistical analysis was performed using the GraphPad Prism software, version 8.0 (San Diego, CA, USA). The statistical significance of mean values for multiple comparisons was monitored in control flies and further assessed using one-way ANOVA with Tukey’s post hoc test. The results were considered significant when *p* < 0.05 (* *p* < 0.05, ** *p* < 0.01, *** *p* < 0.001, **** *p* < 0.0001) and ns for *p* > 0.05.

## 3. Results

### 3.1. Determination of Developmental Parameters of D. melanogaster

**Figure 1:**
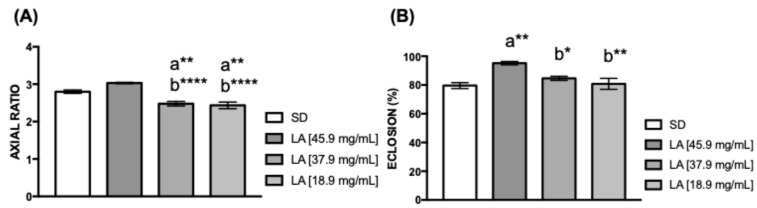
Quantification of pupal volume and eclosion percentage. (**A**) The body shape of a pupa can be described by length (L)/width (W) (*n* = 40). (**B**) Percentage of pupae eclosed (*n* = 40). The values represent the mean ± SEM of five experiments. The results were considered statistically significant when * *p* < 0.05, ** *p* < 0.01, **** *p* < 0.0001: ^a^ vs. SD, ^b^ vs. LA [45.9 mg/mL].

To examine how varying concentrations of linoleic acid (LA) added to a standard diet (SD) influence *D. melanogaster* development (Figure S1), two additional key experiments were conducted. The first measured pupal volume, revealing a decrease in flies fed with 37.9 and 18.9 mg/mL of linoleic acid (** *p* < 0.01, **** *p* < 0.0001) (Figure 1A). The second experiment showed a significant increase in the eclosion rate of flies exposed to 45.9 mg/mL (** *p* < 0.01) (Figure 1B).

### 3.2. Effect of TreadWheel on the Weight of D. melanogaster

**Figure 2:**
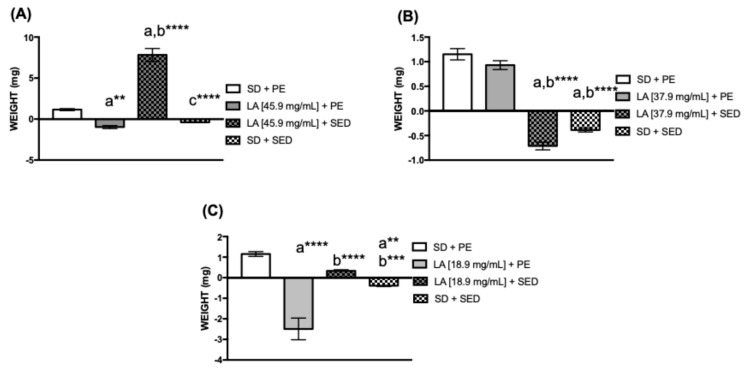
Weight gain in female submitted to diet supplementation with linoleic acid (LA). (**A**) [45.9 mg/mL], (**B**) [37.9 mg/mL], and (**C**) [18.9 mg/mL] with physical exercise (PE) or without exercise stimulus (sedentary or inactive, SED) for 15 days (*n* = 80). The values represent the mean ± SEM of five experiments. The results were considered statistically significant when ** *p* < 0.01, *** *p* < 0.001, **** *p* < 0.0001: ^a^ vs. SD + PE, ^b^ vs. LA + PE, ^c^ vs. LA + SED.

Diet, alone or combined with physical exercise, can alter the animal’s weight, leading to weight gain or loss. To assess weight changes between the groups, female *D. melanogaster* fed with a diet supplemented with 37.9 mg/mL of LA and kept sedentary showed a reduction in weight after 15 days of the experiment compared to flies with physical exercise and LA supplementation (**** *p* < 0.0001) (Figure 2B). However, flies fed with a diet supplemented with 37.9 mg/mL LA combined with physical exercise demonstrated an increase in weight (Figure 2B).

### 3.3. Lactate Content

**Figure 3:**
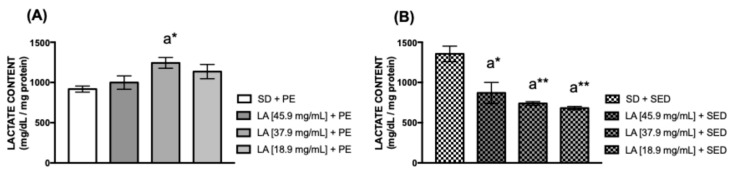
Lactate concentration in (**A**) the thorax of females fed with linoleic acid (LA) and physical exercise (PE), and (**B**) the thorax of female flies supplemented with linoleic acid without PE for 15 days (*n* = 50). The values represent the mean ± SEM of three experiments. The results were statistically significant when * *p* < 0.05, ** *p* < 0.01: ^a^ vs. SD.

Lactate is a molecule with multiple roles in the body, including serving as an energy source, a substrate for pyruvate production, and initiating the Krebs Cycle. Additionally, it promotes cellular tropism, which is enhanced through physical exercise, especially vigorous exercise [41,42,43]. To measure the lactate levels in flies, the diet supplemented with 37.9 mg/mL of linoleic acid combined with physical exercise increased the lactate content (* *p* < 0.05) (Figure 3A). However, flies fed with 45.9 mg/mL (* *p* < 0.05), 37.9 mg/mL (** *p* < 0.01), and 18.9 mg/mL (** *p* < 0.01) of LA and remaining sedentary showed a reduction in lactate levels in the thoraces (Figure 3B).

### 3.4. Glycogen and Cholesterol Levels

**Figure 4:**
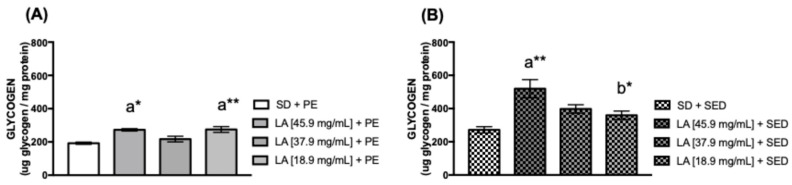
**(A)** Glycogen concentration in the thorax of females fed with linoleic acid and physical exercise (PE), and (**B**) female flies’ thorax supplemented with linoleic acid without PE for 15 days (*n* = 50). The values represent the mean ± SEM of three experiments. The results were considered statistically significant when * *p* < 0.05, ** *p* < 0.01: ^a^ vs. SD, ^b^ vs. LA [45.9 mg/mL] + SED.

**Figure 5:**
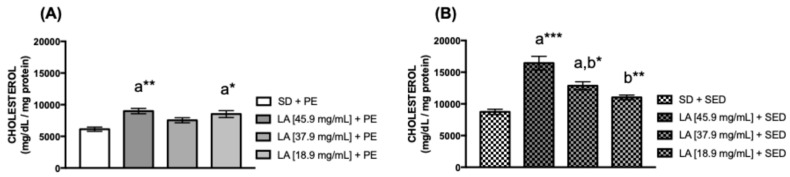
Cholesterol concentration in the thorax of females fed with (**A**) linoleic acid and physical exercise (PE), and (**B**) female flies’ thorax supplemented with linoleic acid without PE for 15 days (*n* = 50). The values represent the mean ± SEM of three experiments. The results were considered statistically significant when * *p* < 0.05, ** *p* < 0.01, *** *p* < 0.001: ^a^ vs. SD, ^b^ vs. LA [45.9 mg/mL] + SED.

Glycogen is a glucose polymer that serves as an intracellular energy reserve found in greater amounts in mammalian hepatocytes and myocytes [44,45]. To evaluate the presence of glycogen and cholesterol in the thorax of female flies, we found that in the exercised group, the association of PE and a diet supplemented with 45.9 mg/mL (* *p* < 0.05) and 18.9 mg/mL (** *p* < 0.01) of LA showed a significant increase in the glycogen concentration (Figure 4A). The same effect was observed in sedentary flies fed with 45.9 mg/mL (** *p* < 0.01) of LA (Figure 4B). Cholesterol is a lipophilic molecule that plays a key role in maintaining the fluidity of the cell membrane [46,47]. Cholesterol levels were higher in flies fed with 45.9 mg/mL (** *p* < 0.01) and 18.9 mg/mL (* *p* < 0.05) of LA with PE (Figure 5A). However, sedentary flies fed with 45.9 mg/mL (*** *p* < 0.001) and 37.9 mg/mL (* *p* < 0.05) of LA showed an increase in cholesterol levels (Figure 5B) compared to untreated (control) flies.

### 3.5. Hydrogen Peroxide and Nitric Oxide Formation

**Figure 6:**
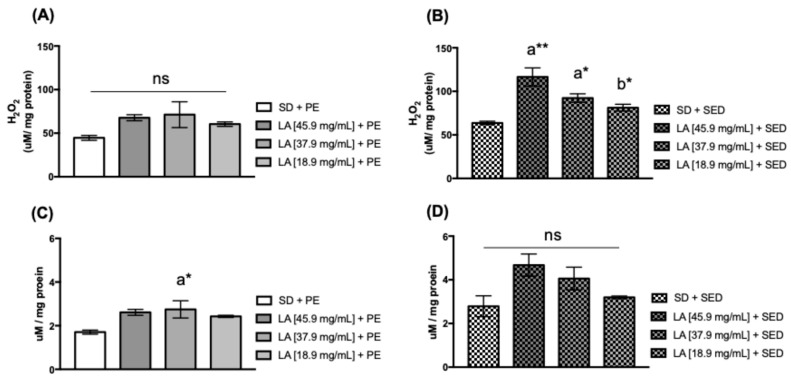
Levels of H_2_O_2_ and ^•^NO in the thorax of females. Flies fed with (**A**,**C**) linoleic acid (LA) and physical exercise (PE), and (**B**,**D**) female flies’ thorax supplemented with linoleic acid without PE for 15 days (*n* = 50). The values represent the mean ± SEM of three experiments. The results were considered statistically significant when * *p* < 0.05, ** *p* < 0.01: ^a^ vs. SD, ^b^ vs. LA [45.9 mg/mL] + SED and ns for *p* > 0.05.

Hydrogen peroxide (H_2_O_2_) is a free radical produced in the electron transport chain; its excess production is associated with inflammation and adaptations generated by physical exercise [48,49,50]. Nitric oxide (**^•^**NO) is a gas that exerts a vasodilator effect, influences immune responses, and acts as a cellular signal. Its production can be stimulated through physical exercise [51,52]. The sedentary group of flies fed with 45.9 mg/mL (** *p* < 0.01) and 37.9 mg/mL (* *p* < 0.05) of LA showed a significant increase in H_2_O_2_ production (Figure 6B). However, trained flies fed with 37.9 mg/mL (* *p* < 0.05) of LA showed an increase in **^•^**NO in the thoraces of trained flies (Figure 6C). No significant difference was detected in the H_2_O_2_ and **^•^**NO production among trained (Figure 6A) and sedentary flies (Figure 6D).

### 3.6. Acetylcholinesterase and Citrate Synthase Activities

**Figure 7:**
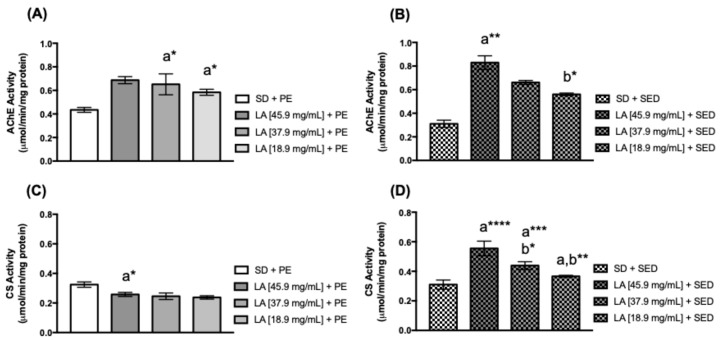
AChE and CS activities in the female flies’ thorax. Flies fed with (**A**,**C**) linoleic acid and physical exercise (PE), and (**B**,**D**) female flies’ thorax supplemented with linoleic acid without PE for 15 days (*n* = 50). The values represent the mean ± SEM of three experiments. The results were statistically significant when * *p* < 0.05, ** *p* < 0.01, *** *p* < 0.001, **** *p* < 0.0001: ^a^ vs. SD, ^b^ vs. LA [45.9 mg/mL] + SED.

Acetylcholinesterase (AChE) is an enzyme that catalyzes the breakdown of acetylcholine (ACh) into acetate and choline. ACh is a powerful neurotransmitter that releases calcium ions (Ca^2+^), which are essential for muscle contraction [39,49,50,51,52]. Trained flies fed 45.9 mg/mL (* *p* < 0.05) of LA showed an increase in the AChE activity (Figure 7A). However, untrained (or sedentary) flies fed with 45.9 mg/mL (**** *p* < 0.0001), 37.9 mg/mL (*** *p* < 0.001), and 18.9 mg/mL (** *p* < 0.01) of LA exhibited a higher AChE activity compared to untreated flies (Figure 7B).

Citrate synthase (CS) is the first enzyme of the Krebs cycle that catalyzes the condensation of acetyl-CoA into citrate. Its activity is related to mitochondrial function and can increase ATP production [19,53,54,55,56,57,58]. CS activity decreased significantly in trained flies fed with 37.9 mg/mL (* *p* < 0.05) and 18.9 mg/mL (* *p* < 0.05) of LA (Figure 7C). However, CS activity was higher in sedentary flies fed with 45.9 mg/mL (** *p* < 0.01) (Figure 7D).

## 4. Discussion

Diet alone or combined with physical activity can change the animal’s weight, causing either gain or loss. Moreover, consuming PUFAs influences gametogenesis, indicating that weight gain may also affect the flies’ appetite [26,59,60,61,62,63]. In this study, groups of *D. melanogaster* that were trained or untrained and fed a diet supplemented with 37.9 mg/mL of LA exhibited opposing effects on weight, suggesting muscle hypertrophy resulting from physical exercise, as seen in rats [64], mice [65], fish [65], *C. elegans* [66], and flies [67].

In humans, lactate and glycogen are linked to PE and are primarily utilized during moderate to high-intensity exercise. Their levels rise in connection with muscle hypertrophy and function as intracellular energy reserves, which are abundant in mammalian hepatocytes and myocytes [44,45]. In *D. melanogaster*, these compounds are vital for embryonic development, serving as main energy sources from early stages to adulthood, and promoting muscle tropism, especially through vigorous physical activity [37,38,39,68,69,70]. The TreadWheel apparatus involves intense aerobic activity, and our findings indicate that flies trained and fed 37.9 mg/mL of LA had elevated lactate levels compared to untreated controls. Conversely, sedentary flies fed at 45.9 mg/mL, 37.9 mg/mL, or 18.9 mg/mL of LA exhibited lower lactate levels than untreated flies. During high-intensity exercise, lactate rises and can be used for energy when converted to pyruvate in the Krebs Cycle; it also acts as a signaling molecule, stimulating mitochondrial activity [71].

Our data indicate elevated glycogen levels in the thoraces of flies trained and fed with 45.9 mg/mL and 18.9 mg/mL of LA. Additionally, sedentary flies fed 45.9 mg/mL of LA showed high glycogen levels. In mice, a high-fat diet increases liver glycogen by activating mTORC1, which boosts glycogen synthase activity [72]. This suggests that LA intake may directly influence weight gain, as observed in sedentary flies fed with LA [69,73].

Cholesterol is a lipophilic molecule that plays a key role in the production of steroid hormones, the storage of fat-soluble vitamins, and the fluidity of the cell membrane [46,47]. Trained flies fed with a supplemented diet containing 45.9 mg/mL and 18.9 mg/mL of LA influenced the amount of cholesterol, mainly in trained flies. However, sedentary flies showed an increase in cholesterol levels after being fed 45.9 mg/mL and 37.9 mg/mL of LA compared to untreated flies. Studies on physical activity suggest greater membrane fluidity, which favors a “controlled” increase in cholesterol levels compared to those of sedentary individuals, similar to how aerobic exercise alters the lipid profile in humans [9,26,46,47]. In *D. melanogaster*, similar to vertebrates, it requires cholesterol directly from dietary intake as a precursor for steroid hormones and as a structural component of cell membranes [42]. In addition, physical exercise also alters lipid levels, characterized by reduced triglyceride and glycerol levels [24,74,75].

Hypercholesterolemia in humans causes harmful health effects, such as atherosclerosis and tissue inflammation, which in turn increase the production of free radicals, including hydrogen peroxide (H_2_O_2_) [10,76,77,78] produced in the electron transport chain; its excess production is associated with inflammation and adaptations generated by physical exercise [48,49,50]. In physical exercise, H_2_O_2_ is linked to exercise-induced adaptations, promoting greater resistance to oxidative stress, enhancing muscle antioxidant capacity, and boosting mitochondrial activity [48,49,50,79,80]. When measuring H_2_O_2_, we observed an increase in it among sedentary animals that consumed 45.9 and 37.9 mg/mL of LA compared to untreated flies, suggesting a pro-oxidant effect of the diet with linoleic acid [34,81,82]. This finding can be justified by the propensity of PUFAs to non-enzymatic (autooxidation and photooxidation) and enzymatic oxidation, promoting oxidative stress mainly by increasing the concentration of lipid peroxidation aldehydes and activating superoxide anion generators, such as NADPH oxidases [79].

The oxidative stress caused by increased oxygen consumption in PE can trigger another adaptation: an elevation in nitric oxide (^•^NO), a gas that acts as a vasodilator and is also increased during immune responses, where it serves as a cell signaling molecule [51,52,83]. In this study, no significant difference was detected in **^•^**NO production among sedentary flies. However, flies trained and fed with 37.9 mg/mL of LA showed an increase in ^•^NO, suggesting this may be related to increased adipose tissue, which could enhance phagocytic activity due to its inflammatory nature. Yet, the absence of **^•^**NO changes in sedentary groups does not exclude **^•^**NO-dependent mechanisms, as **^•^**NO is highly dose- and context-dependent. Previous work has shown that very low-dose dietary nitrite can elevate **^•^**NO signaling and modulate nutrient sensing in *Drosophila melanogaster* [80].

In humans, aerobic physical exercise affects citrate synthase (CS) activity [27,58,81,82]. CS is the first enzyme of the Krebs cycle that catalyzes the condensation of acetyl-CoA into citrate, located in the mitochondrial intermembrane space, and its activity is connected to mitochondrial function [19,56,57]. Our results showed that trained flies fed 37.9 mg/mL and 18.9 mg/mL of LA exhibited lower CS activity, suggesting that the trained flies used ATP produced through glycolysis independently of mitochondria, supplying energy for faster muscle contractions and consequently resulting in lactate buildup. Conversely, sedentary flies fed 45.9 mg/mL of LA exhibited higher CS activity because their thoracic muscles did not require a high level of energy.

The muscle contraction mechanism is influenced by several factors, including ATP production and the cholinergic system [83]. Acetylcholine (ACh) concentration is regulated by acetylcholinesterase (AChE), which breaks down ACh into acetate and choline. ACh is a powerful neurotransmitter that releases calcium ions, which are essential for muscle contraction [39,53,54,55,56]. In this study, the intake of 45.9 mg/mL of LA indicates an improvement in the cholinergic system in both trained and sedentary flies, which are involved in calcium uptake for muscle contraction. In contrast, the central nervous system maintains a balance between neurotransmitters and ACh [84], enhancing locomotion in *D. melanogaster* [19,20,85,86,87,88].

Finally, based on this study, we propose using *D. melanogaster* in the TreadWheel as a model for physical exercise, with a diet supplemented with linoleic acid, which shows developmental and biochemical changes, including increased eclosion rate, weight gain, enhanced acetylcholinesterase activity, and reduced lactate and cholesterol accumulation. However, sedentary flies exhibited significant changes in factors such as glycogen and cholesterol levels, as well as acetylcholinesterase and citrate synthase activities. In this study, we demonstrated, for the first time, that both trained and sedentary flies fed with linoleic acid show improvements in behavioral and biochemical parameters. Additionally, these results encourage further research and investigation into the potential mechanisms involved in physical exercise and diet supplemented with linoleic acid, also in association with antioxidant supplementation, with the aim of minimizing the potential pro-oxidant and auto-oxidation effects of PUFAs.

## 5. Conclusions

*D. melanogaster* fed with linoleic acid and trained using a TreadWheel system resulted in an improvement in biochemical parameters, including an increase in weight, lactate, glycogen, cholesterol, nitric oxide levels, and acetylcholinesterase activity. In this study, we demonstrated, for the first time, that the ingestion of linoleic acid combined with physical exercise produces a synergistic effect that induces significant changes in flies, suggesting a potential modulation of mitochondrial and cholinergic pathways by linoleic acid and implying neuromuscular activity that warrants further studies in vertebrate models.

## Data Availability

The datasets generated and/or analyzed during this study are available from the corresponding author upon reasonable request.

## Supplementary Materials

The following supporting information can be downloaded at: https://www.mdpi.com/article/10.3390/biology15080607/s1, Figure S1: Effect of diet supplemented with linoleic acid and diet plus exercise on *D. melanogaster* survival rate. (**A**) flies with five days, (**B**) flies with ten days, and (**C**) flies with fifteen days old after eclosion (*n* = 120). The values represent the mean ± SEM of five experiments.
