# Supplementary material for: Effect of Aerobic Exercise and a Diet Supplementation with Linoleic Acid on Metabolic Parameters in Drosophila melanogaster
Source: Biology (Basel). 2026 Apr 12;15(8):607. doi: 10.3390/biology15080607 (PMC13113448; doi:10.3390/biology15080607)
Supplement: Supplementary file 1 [file biology-15-00607-s001.zip › biology-4154004-supplementary.pdf]

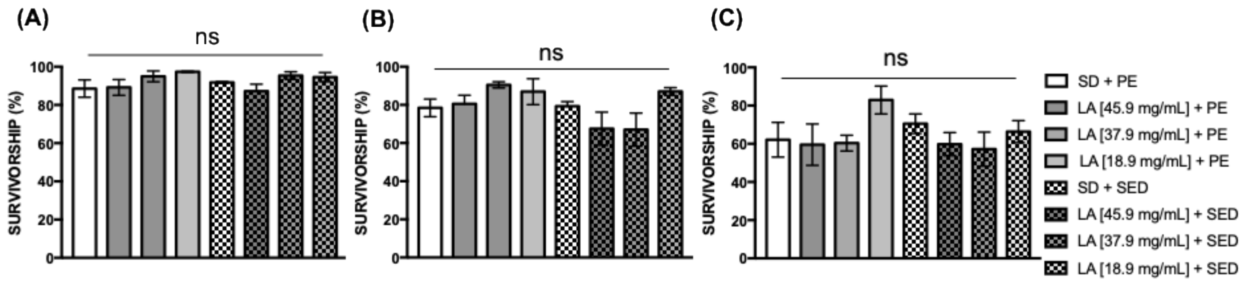

Figure S1: Effect of diet supplemented with linoleic acid and diet plus exercise on *D. melanogaster* survival rate. (**A**) flies with five days, (**B**) flies with ten days, and (**C**) flies with fifteen days old after eclosion (*n* = 120). The values represent the mean ± SEM of five experiments.
